# A NODding acquaintance with ER stress

**DOI:** 10.1038/cddiscovery.2016.37

**Published:** 2016-05-30

**Authors:** CA Stafford, U Nachbur

**Affiliations:** 1 The Walter and Eliza Hall Institute of Medical Research, 1G Royal Parade, Parkville, VIC 3052, Australia; 2 Department of Medical Biology, University of Melbourne, Melbourne, VIC 3010, Australia

Accumulation of unfolded or misfolded proteins in the lumen of the endoplasmatic reticulum (ER) results in ER stress and induces the unfolded protein response (UPR). The UPR consists of distinct cellular processes, such as increased transcription of repair proteins and chaperones, cell death and the induction of an inflammatory response resulting in the expression of pro-inflammatory cytokines such as IL-6. In a recent issue of *Nature*, Keestra-Gounder *et al.*^1^ provided the missing link between ER stress and the activation of NF-*κ*B and cytokine production. The NOD1/2 inflammatory pathway, classically associated with intracellular sensing of bacterial peptidoglycan (PGN), was shown to be required for ER stress-induced cytokine production. This work paves the way for new treatment strategies of inflammatory diseases that are triggered by ER stress.

Unfolded or misfolded proteins are normally sensed by a robust quality-control system and, if they are unable to be corrected, sent for degradation using the introduced above ER-associated degradation pathway (ERAD). Prolonged UPR is introduced above UPR activates three ER stress sensors: double-stranded RNA-activated protein kinase-like ER kinase (PERK), activating transcription factor 6 (ATF6) and the inositol requiring kinase 1*α* (IRE1*α*; Figure 1). Activation of these sensors results in increased degradation of misfolded proteins and in the induction of transcription of ERAD components and protein-folding chaperones to reduce stress on the ER.

In addition to the efforts to reduce pressure on the ER, PERK is able to induce cell death and IRE1*α* can trigger an inflammatory response. When misfolded proteins accumulate in the ER, PERK oligomerizes and activates the transcription factor C/EBP homologous protein, resulting in the activation of pro-apoptotic members of the Bcl2 family, such as Puma and Bim.^2^ On the other hand, IRE1*α* activation recruits TNF receptor-associated factor 2 (TRAF2), which results in the activation of the NF-*κ*B and JNK pathways and in the production of inflammatory cytokines.^3^

In their recent work, the Bäumler and Tsolis groups showed that cells and mice deficient in the TRAF2–NOD1/2–RIPK2 axis are deficient in IRE1*α-*induced IL-6 production.^1^ Genetic loss of the pattern recognition receptors NOD1/2 or receptor interacting kinase 2 (RIPK2) resulted in reduced IL-6 production *in vivo* and *in vitro* upon treatment with the ER stress inducer thapsigargin. Loss of NOD1/2 or RIPK2 had the same effect as treatment with KIRA6, an allosteric kinase inhibitor of IRE1. Deletion of NOD1/2 solely reduced signaling downstream of IRE1*α*, whereas signaling from ATF6 and PERK was not affected. These results show the role of NOD1/2 in the IRE1*α* arm of the ER stress response and raise the possibility of therapeutic intervention via the NOD pathway to reduce ER stress-induced inflammatory responses. Further support for these observations can be drawn from the similarity of cytokine profiles after induction of either ER stress or peptidoglycan (PGN) stimulation, both resulting in high IL-6 and IL-1*β* expression and in a Th17-driven immune response.^4,5^

NOD1 is known as an intracellular sensor of the PGN component *γ*-D-glutamyl-meso-diaminopimelic acid present in the cell wall of all Gram-negative and a few Gram-positive bacteria,^6^ whereas NOD2 is activated by muramyl dipeptide (MDP) present in PGN of all Gram-positive and -negative bacteria.^7^ Regions in the leucine-rich repeat (LRR) of NOD1 and NOD2 have been identified to be crucial for its activation by PGN;^8^ yet there is controversy concerning whether DAP and MDP bind directly to NOD1 or NOD2. DAP has been shown to bind to the LRR of NOD1, yet with a relatively high *K*(d) value of 34.5 *μ*M,^9^ questioning its relevance in a cellular setting.

The new work of Keestra-Gounder *et al.*^1^ is a second report of PGN-independent activation of the NOD signaling pathway, thereby challenging the direct activation of NOD receptors by PGN. The group previously described the capacity of NOD1 to sense the activity status of small Rho GTPases to activate NF-*κ*B in a RIPK2-dependent manner.^10^ A crucial adaptor for NOD activation seems to be TRAF2, which oligomerizes with IRE1*α* upon its activation; however, it is not yet clear how TRAF2 can activate NOD1/2. The mode of activation of NOD receptors is, therefore, still not clearly resolved and will require further clarification.

Somewhat surprising is the fact that both NOD1 and NOD2 were required for NF-*κ*B activity downstream of IRE1*α*. Loss or inhibition of either one of NOD1 or NOD2 does not influence signaling via the remaining NOD receptor after stimulation with DAP or MDP, respectively. However, in the case of activation via IRE1*α*, both NOD receptors seem to be required for NF-*κ*B activation, suggesting a function as a heterodimer. The absolute requirement of RIPK2 is consistent with the current knowledge of its essential role in signaling downstream of NOD1 and NOD2.

To show *in vivo* relevance for their findings, Keestra-Gounder *et al.* injected mice with *Brucella abortus*, a bacterial pathogen that causes premature abortion in cattle. *B. abortus* infection induces ER stress by injecting the virulence factor VceC into host cells, which translocates to the ER and disrupts its normal function. Infection of mice with *B. abortus,* but not with a *vceC* mutant strain, induced IRE1*α*-dependent IL-6 production, which was significantly reduced in NOD1/2-deficient mice or when mice were treated with the ER stress inhibitor TUDCA or with the IRE1*α* kinase inhibitor KIRA6. Whereas disruption of the IRE1*α*–NOD–RIPK2 axis reduced the inflammatory phenotype and the abortion rate, bacterial load in *B. abortus*-infected mice remained unchanged.

The results of Keestra-Gounder *et al.* not only indicate that ER-stress-driven inflammation is at least partially responsible for the main pathological outcomes of *B. abortus* infection, but also show that reducing the inflammatory response is sufficient to alleviate the key symptoms. This could be relevant for a range of human inflammatory conditions where we know that ER stress is involved, such as Crohn’s disease, rheumatoid arthritis, type 2 diabetes or multiple sclerosis (MS).^11^ Intriguingly, the NOD–RIPK2 pathway has been connected to all of these diseases, and the link between ER stress and NOD signaling might open new avenues for their treatment. RIPK2 inhibitors have already shown promise in the treatment of experimental autoimmune encephalomyelitis (EAE), the murine model for MS.^12^ Whether this effect was because of inhibition of the ER stress response during EAE or the inhibition of PGN-induced RIPK2 activation remains to be determined.

The findings of Keestra-Gounder *et al.* are certainly exciting, and future research will tell whether inhibition of the NOD–RIPK2 pathway can be exploited for the treatment of ER-stress-driven inflammatory diseases, as suggested in Figure 1. However, we should keep in mind that all of these diseases have complex etiologies and, as in cancer therapy, the key to successful treatment of inflammatory diseases might lie in combination therapies.

On a molecular level, the mechanistics of NOD activation will have to be revisited. Overexpression studies in HEK293T cells have brought valuable insights into potential factors and mechanisms of innate immune signaling; however, it is now time to explore and determine the role of these proteins at endogenous levels and in relevant cell types.

## Figures and Tables

**Figure 1:**
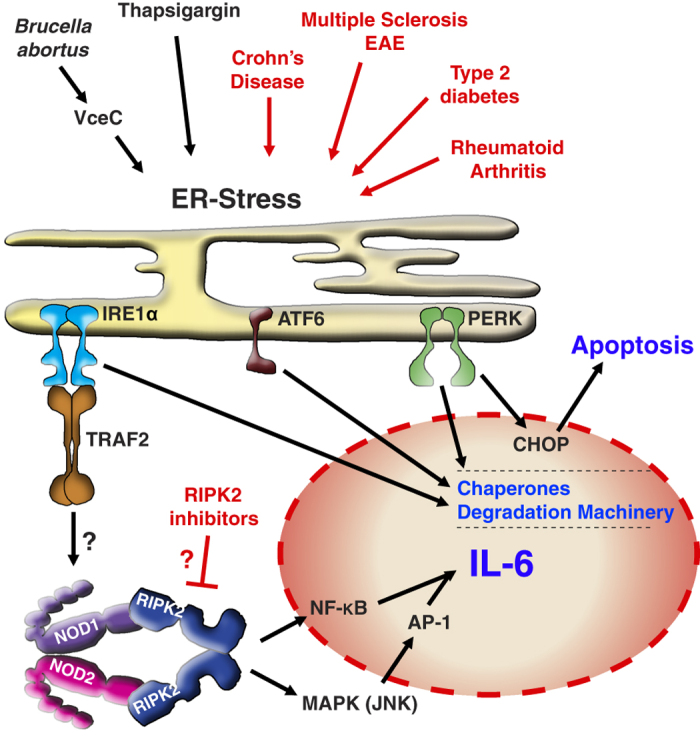
Schematic of findings and potential applications from Keestra-Gounders *et al.* ER-stress inducers used in the study of Keestra-Gounders and colleagues (black) and ER-stress-associated diseases (red) induce three ER-localized receptors. The activation of IRE1*α*, ATF6 and PERK results in transcriptional upregulation of the degradation machinery and chaperones to reduce the pressure on the ER. In addition, PERK induces cell death via transcriptional upregulation of C/EBP homologous protein (CHOP), and IRE1*α* oligomerization results in the recruitment of TRAF2, which activates the NOD1/2–RIPK2 pathway, resulting in activation of NF-*κ*B and AP-1 and the transcription of inflammatory cytokines such as IL-6. This newly described pathway suggests that inhibition of the NOD–RIPK2 axis, for example by RIPK2 kinase inhibitors, might be a strategy to reduce inflammation in ER-stress-driven diseases.
